# Simple and customizable method for fabrication of high-aspect ratio microneedle molds using low-cost 3D printing

**DOI:** 10.1038/s41378-019-0088-8

**Published:** 2019-09-09

**Authors:** Kevin J. Krieger, Nicky Bertollo, Manita Dangol, John T. Sheridan, Madeleine M. Lowery, Eoin D. O’Cearbhaill

**Affiliations:** 1 UCD Centre for Biomedical Engineering, University College Dublin, Belfield, Dublin 4 Ireland; 2 School of Mechanical & Materials Engineering, University College Dublin, Belfield, Dublin 4 Ireland; 3 School of Electrical & Electronic Engineering, University College Dublin, Belfield, Dublin 4 Ireland

**Keywords:** Engineering, Nanoscience and technology

## Abstract

We present a simple and customizable microneedle mold fabrication technique using a low-cost desktop SLA 3D printer. As opposed to conventional microneedle fabrication methods, this technique neither requires complex and expensive manufacturing facilities nor expertise in microfabrication. While most low-cost 3D-printed microneedles to date display low aspect ratios and poor tip sharpness, we show that by introducing a two-step “Print & Fill” mold fabrication method, it is possible to obtain high-aspect ratio sharp needles that are capable of penetrating tissue. Studying first the effect of varying design input parameters and print settings, it is shown that printed needles are always shorter than specified. With decreasing input height, needles also begin displaying an increasingly greater than specified needle base diameter. Both factors contribute to low aspect ratio needles when attempting to print sub-millimeter height needles. By setting input height tall enough, it is possible to print needles with high-aspect ratios and tip radii of 20–40 µm. This tip sharpness is smaller than the specified printer resolution. Consequently, high-aspect ratio sharp needle arrays are printed in basins which are backfilled and cured in a second step, leaving sub-millimeter microneedles exposed resulting microneedle arrays which can be used as male masters. Silicone female master molds are then formed from the fabricated microneedle arrays. Using the molds, both carboxymethyl cellulose loaded with rhodamine B as well as polylactic acid microneedle arrays are produced and their quality examined. A skin insertion study is performed to demonstrate the functional capabilities of arrays made from the fabricated molds. This method can be easily adopted by the microneedle research community for in-house master mold fabrication and parametric optimization of microneedle arrays.

## Microfabrication: Low-Cost 3D Printing Method for Producing Microneedle Molds

A low-cost, simple, customizable method has been developed for fabricating microneedle molds by means of a commercially available stereolithography 3D printer. Microneedle arrays (MNAs) are devices composed of micron-sized needles that allow the transfer of compounds, such as drugs, across the outer layer of a tissue, usually the skin. Conventional methods to fabricate the microneedles for such MNAs demand complex, expensive manufacturing facilities and expertise in microfabrication. However, a team headed by Kevin Krieger in Dr. Eoin O’Cearbhaill’s Medical Device Design Group at University College Dublin, Ireland was able to develop a simple fabrication method using a commonly available, affordable 3D printer that allows rapid production of microneedle master molds with easily customizable needle parameters and demands no microfabrication expertise. The authors believe that their method can be readily adopted by researchers for master mold fabrication and parametric optimization of MNAs.

## Introduction

Microneedles arrays (MNAs) are devices comprised micron-sized needles which allow for transfer of a compound or signal across the outer layer of tissue, typically the skin. Owing to their short height, they are considered painless and can be considered a minimally invasive device. Applications of microneedle patches include transdermal drug delivery (vaccines^1^, insulin^2^), chemical biosensing (glucose^3^, DNA biomarkers^4^), electrical biosignal recording (electrocardiography^5^, surface electromyography^6^, intramuscular electromyography^7^, electroencephalography^8^), electrical stimulation (electrotactile display^9^), and as neural interfaces^10^. Depending on the requirement, microneedles may be solid^11^, coated^12^, hollow^13^, porous^14^, hydrogel based^15^/swellable^16^, or of a merged-tip geometry^17^. There are many materials which have been utilized in microneedle research, including silicon, glass, ceramics, metals, solid polymers, as well as swellable hydrogel polymers. Given the vast amount of applications and materials that may be used, cost-effective methods for the fabrication of microneedles for research and development purposes are needed.

There are several important design criteria to consider when designing and fabricating microneedles. Limiting the height of a needle to the sub-millimeter range allows for microneedle insertion to remain largely painless^18^. Needle height also controls the depth to which a drug/vaccine may be delivered, or the depth from which a compound/signal is extracted in sensing applications. The aspect ratio of the needle influences ease of insertion and mechanical integrity. While higher aspect ratio needles are easier to insert, a lower aspect ratio results in mechanically stronger needles. In drug delivery applications, both height and aspect ratio govern individual microneedle volume and therefore control dosage. The tip radius is also a key parameter for ensuring microneedle skin insertion. Control of these dimensional parameters of microneedles is important, as this allows for tailoring of microneedle functionality.

Microneedle arrays may be fabricated for direct application or for use as a master for microneedle mold fabrication. The latter approach allows for subsequent replica molding of MNAs from a wide range of materials and lends itself well to device optimization in a laboratory setting.

Commonly, microelectromechanical systems fabrication techniques are applied in the fabrication of microneedles, especially for MNA masters for subsequent mold making. Methods used are chemical wet etching^19^, ultraviolet (UV) lithography^20^, and deep reactive-ion etching^21^. Other fabrication techniques include electrical discharge machining^22^, direct laser micromachining^23^, and micromilling^24^. These methods can produce microneedles with excellent microscale features. However, these methods also require either expensive specialized equipment and/or advanced manufacturing facilities such as clean rooms. Alternative microneedle fabrication methods have been investigated, including drawing lithography (thermal^25^, electro-^26^, magnetorheological^27^, UV^28^, air blowing^29^) and centrifugal lithography^30^.

Laboratories focused on microneedle research, however, often do not have the facilities in-house, know-how nor desire to invest time to fabricate their own custom microneedle structures. General engineering workshops may not be readily available to them, especially for researchers from outside the field of engineering, and therefore these researchers commonly buy in microneedle molds. Replica molding offers the advantage that it is inexpensive, quick, repeatable, simple, up-scalable and usable with a wide range of thermoplastic, thermoset and swellable/hydrogel-forming polymers, as well as ceramics and metals. However, in the research setting, making molds or buying molds is relatively expensive, especially when specific microneedle parameters, such as microneedle height, aspect ratio, array size, and needle-to-needle distance may be subject to change, thus requiring buying in of multiple molds which is both cost- and time-prohibitive. One approach for in-house microneedle mold fabrication is replica molding of off-the shelf products, such as acupuncture needles^6^ or commercial microneedle patches (e.g., Derma Stamp^31^). Acupuncture needle replica molding, however, can be time-consuming while commercial microneedle patch replica molding limits customizability. Another approach to fabricate low-cost microneedle molds which allows for freedom of design was proposed by Nejad et al.^32^. Their method enables microneedle female mold fabrication by means of CO_2_ laser ablation of an acrylic sheet. While this is a subtractive manufacturing technique, a promising alternative emerging area within microneedle fabrication is additive manufacturing (AM).

AM can be simple, easily customizable, cost-effective, and produces less waste compared with many subtractive manufacturing techniques and various methods of AM have been employed in the fabrication of microneedle arrays. AM can be classified into seven general categories according to ISO standards (ISO/ASTM 52900:2015) which are binder jetting, directed energy deposition, material extrusion, material jetting, powder be fusion, sheet lamination, and vat photopolymerization^33^.

A relatively user-friendly AM technique is material extrusion-based fused filament fabrication (FFF), also referred to as fused deposition modeling (FDM), which has the advantage that it is affordable and simple-to-use systems are readily available. AM has been used to easily fabricate more complex shaped needles, such as honeybee-inspired microneedles^34^, angled microneedles^35^, and arrowhead-shaped microneedles^36^, which may prove difficult to fabricate using conventional fabrication methods. It allows for printing with low-cost biocompatible materials such as polylactic acid (PLA). However, FFF also suffers from poor resolution compared with other 3D printing techniques, and is generally not able to directly produce the sharp tips required for microneedles although a two-step fabrication method has been proposed to overcome the resolution limitations^37^. Material jetting-based AM in which droplets of a photopolymer are dispensed and light-cured to build the part in a layer-by-layer approach has shown to produce better quality needles^38^. Vat photopolymerization-based AM methods in which a liquid photopolymer vat is selectively cured using light also display better resolution compared with FFF. Certain vat photopolymerization-based methods have been shown to directly print very sharp microscale needles, including two-photon polymerization^39,40^ and continuous liquid interface production^36^. However, these methods are costly, not readily available and therefore not convenient for in-house microneedle manufacture by most researchers interested in microneedle applications. Various low-cost vat photopolymerization-based AM methods have also been explored for MNA fabrication, including projection-based direct light processing (DLP)^41–44^ and scanning-based stereolithography (SLA)^45,46^. In DLP, a digital mirror projects each cross-sectional layer which cures the photopolymer and this process is repeated as the part is built in a layer-by-layer approach. Scanning-based SLA is a technology wherein a part is also printed out of a UV-curable resin. As opposed to DLP, where each layer is projected as whole, in SLA a laser tracks and “draws” each layer, curing the resin as it travels along the *x*–*y* plane. Studies to date using these methods, however, have only been able to fabricate MNAs which exhibit some but not all of three key geometric parameters important for high-quality microneedles which are good tip sharpness, sub-millimeter height and high-aspect ratio.

**Fig. 1:**
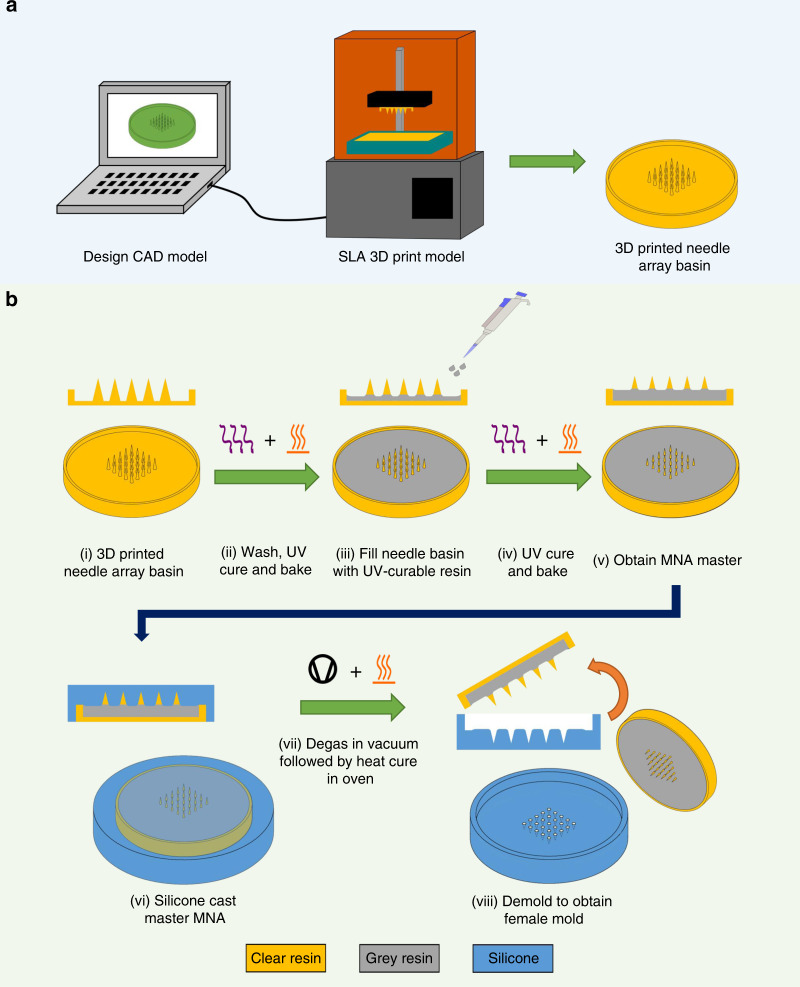
Overview of “Print & Fill” fabrication method. **a** Needle array basin design followed by 3D printing of the design using a Form 2 SLA printer. **b** MNA master mold fabrication method (i) take 3D printed needle array basin; (ii) washing followed by UV curing and baking of printed needle array basin; (iii) filing of needle array basin with UV-curable resin; (iv) second UV curing and baking; (v) obtain MNA master; (vi) silicone casting of MNA master; (vii) silicone mold is degassed followed by heat cure in oven; (viii) demolding to obtain usable microneedle mold

In this study, we present a novel customizable method for fabrication of microneedle masters in the research setting, which may be used to produce female molds which aims to overcome limitations of previous microneedles produced using low-cost desktop 3D printers which often suffer from low tip radius and/or low aspect ratios. We first conducted a parametric study, examining the printer’s capabilities of printing needle structures with sufficient tip sharpness to pierce the skin. To improve feature resolution, we developed a two-step “Print & Fill” technique which allows for the indirect fabrication of customizable microneedle masters for mold making using SLA 3D printing (Fig. 1). Subsequently, microneedle masters and molded microneedle arrays are dimensionally characterized and functionally assessed for reproducibility.

## Results

### Parametric study

First, we conducted a study on printing needles using a desktop SLA 3D printer (Form 2, Formlabs Inc., Somerville, MA, USA) and investigated how to optimize settings to produce best possible needle geometry. The parametric study investigated the effects of varying print settings and needle design and quantified print success by measuring four parameters of the printed needles (needle height, needle angle, tip radius, and straightness of the needle).

### Layer height

**Fig. 2:**
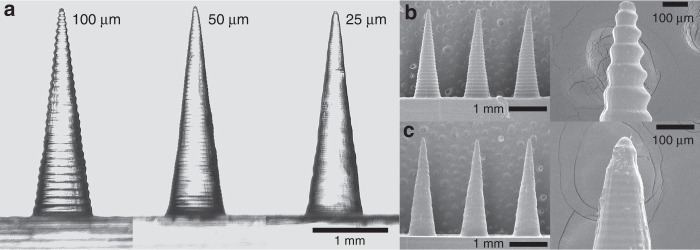
Effect of layer height on print quality. Images of input height 3000 µm and aspect ratio 4:1 needles printed at different layer heights. **a** Comparison of 10, 50, and 25 -µm layer height needles; SEM images of **b** 100 -µm; and **c** 25 -µm layer height needles

The printer allows for setting the individual layer height during print to 25, 50, and 100 µm (thickness of each layer in the *z*-direction). A layer height of 25 µm results in a longer print as the virtual model is divided up into more individual layers to be printed. We printed needles at an aspect ratio of 4:1 to see the effect of layer height on the print quality. The smaller the layer height, the smoother the needle surface becomes as can be seen in Fig. 2.

**Fig. 3:**
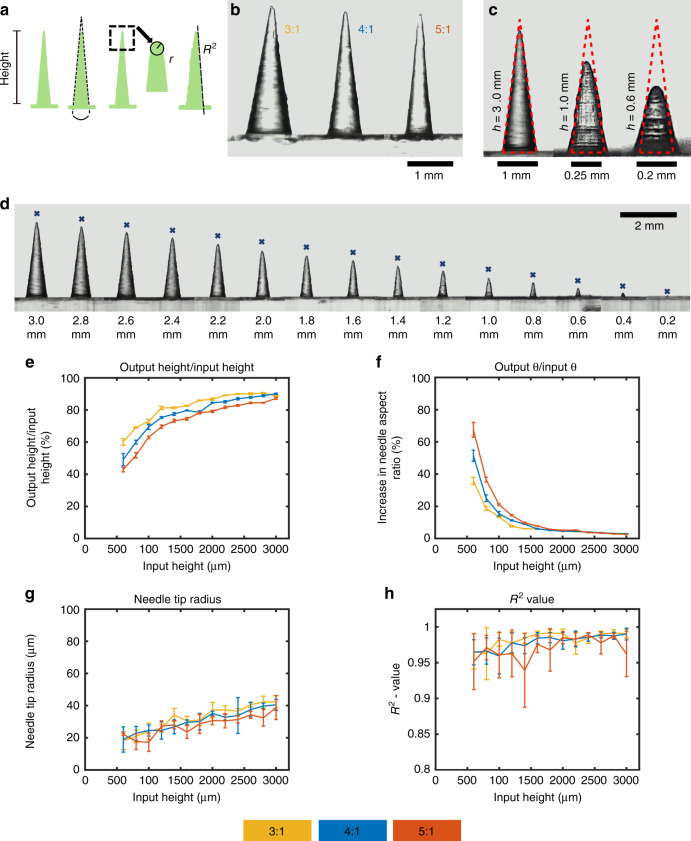
Parametric study of printed needles. **a** Four parameters which were investigated: needle height, needle angle *θ*, tip radius, and needle straightness as determined by the *R*^2^ value; **b** needles with input height of 3 mm and input aspect ratio of 3:1, 4:1, and 5:1 (left to right); **c** input 4:1 aspect ratio needles of input height 3, 1, and 0.6 mm with the theoretical dimensional of the needle outlined; **d** input 4:1 aspect ratio needles with heights ranging from 3 to 0.2 mm (marked by an “x” is the theoretical input height); **e** output-to-input height ratio; **f** output-to-input needle angle *θ* ratio; **g** needle tip radius; **h** *R*^2^ value; (*n* = 5, results displayed as mean ± standard deviation)

Choosing to continue with the 25 -µm layer height print setting as it resulted in smoother needles, the parametric study then examined four design parameters. Needles aligned as 1 × 5 arrays with a 1.5 -mm needle-to-needle distance were printed varying the respective design and print parameters. We examined heights ranging from 3 to 0.2 mm for needle aspect ratios of 3:1, 4:1, and 5:1, the results of which can be seen in Fig. 3.

After printing, arrays were imaged and needles with an input height 200 µm and 400 µm deemed of not of good enough quality to warrant measurements. The remaining needle arrays were then parametrically analyzed.

### Needle height

Printing 1 × 5 arrays with aspect ratios of 3:1, 4:1, and 5:1 resulted in needles of shorter height compared with their respective input values. As can be seen in Fig. 3d, e, the relative difference in output height to input height decreased both with diminishing aspect ratio and increased input height. A printed 3:1 aspect ratio needle with input height of 3000 µm was 11% shorter, while a needle with an input height of 600 µm was over 40% shorter than specified. These relative differences were greater for higher input aspect ratios. For 4:1 aspect ratio needles, the difference was 10% for a 3000 -µm, and 51% for a 600 -µm input height. This further increased for 5:1 aspect ratio needles to 13%, and 57% for a 3000 -µm and a 600 -µm input height, respectively.

### Needle angle

For all three aspect ratio needles, *θ* remained relatively constant until the input needle height was set below 1600 µm as can be seen in Fig. 3f. In that range, the actual *θ* value for both 3:1 and 4:1 aspect ratio needles was −3% ± 2% of the set value. This value was measured to be −1% ± 8% for 5:1 aspect ratio needles. For lower input heights, this value increased rapidly with decreasing input height. For an input height of 600 µm, this value increased to 57, 104, and 153 % for 3:1, 4:1, and 5:1 aspect ratio needles, respectively. The higher the aspect ratio, the greater the deviance from the theoretical angle *θ* was.

### Tip radius

Measuring the tip radii, there appeared to be a general trend for the tip radius to decrease with decreasing input needle height. The tip radii lay between 20 and 40 µm as seen in seen in Fig. 3g.

### Needle straightness

The coefficient of determination *R*^2^ values of lines fitted to the needle edges were generally >0.95 as can be seen in Fig. 3h. This high degree of linearity is indicative of needles with good needle straightness.

### “Print & Fill” microneedle mold fabrication

**Fig. 4:**
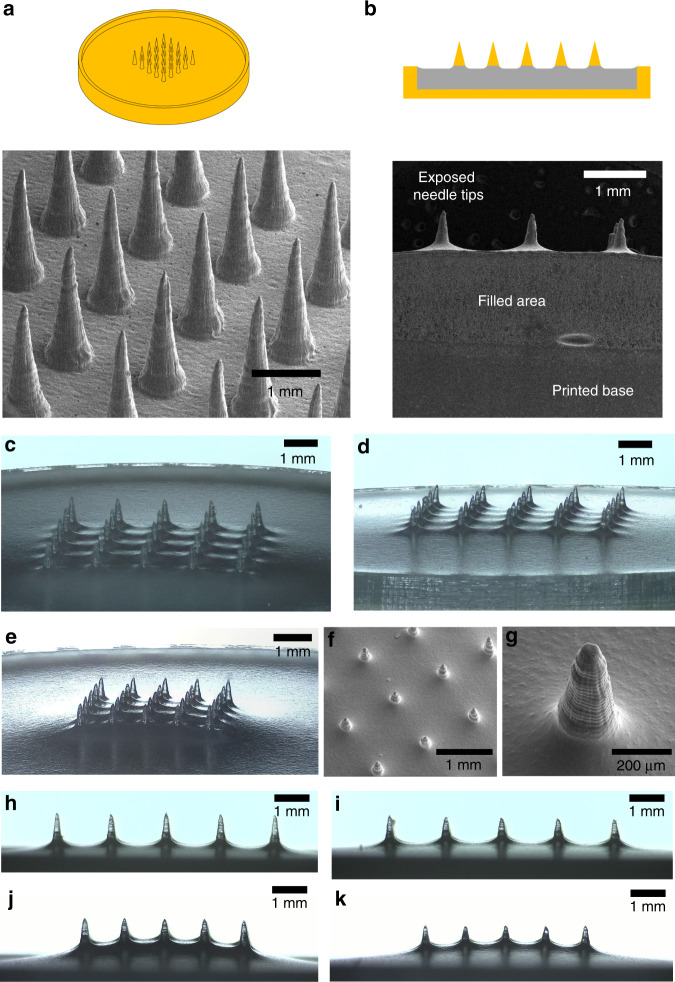
Printed & filled master MNAs (input height: 2.5 mm, aspect ratio: 4:1). **a** CAD drawing and SEM image of 1.5 mm needle-to-needle distance MNA basin before filling; **b** sketch and SEM of laser-cut cross-section of a filled MNA basin with 1.5 mm needle-to-needle distance and a fill weight of 600 mg; **c**, **d** 1.5 mm needle-to-needle distance after filling and curing with a fill weight of 500 mg (**c**) and 600 mg (**d**), respectively; **e** optical image; and **f**, **g** SEM images of printed and filled MNA (needle-to-needle distance: 1 mm, fill weight: 500 mg); **h**, **i** side view of 1.5 mm needle-to-needle distance MNA after filling and curing with a fill weight of 500 mg (**h**) and 600 mg (**i**) respectively; **j**, **k** side view of 1.5 mm needle-to-needle distance MNA after filling and curing with a fill weight of 500 mg (**j**) and 600 mg (**k**) respectively

Optimized settings from the parametric study were used as input for the design of needle array basins. Basins containing an array of 5 × 5 needles with 2.5 -mm input height, 4:1 aspect ratio, and layer height of 25 µm were printed for needle-to-needle distances of 1 mm and 1.5 mm. Basins of both needle-to-needle distances were then filled with a photocurable resin with a fill weight of 500 mg and 600 mg to produce four different types of arrays with needles in the sub-millimeter range (Fig. 4). For 1.5 mm needle-to-needle distance arrays, a fill weight of 500 mg resulted in MNAs with ca. 740 µm needle height (Fig. 4a) while a 600 mg fill weight resulted in needles of ca. 580 µm height (Fig. 4b). For arrays with 1 mm needle-to-needle distance, a 500 mg fill weight resulted in 560 µm (Fig. 4c, e–g) and a 600 mg fill weight resulted in 420 µm (Fig. 4d) height needles. Height was measured from the tip to the onset of the meniscus.

Subsequently, fabricated MNAs were used to make silicone microneedle molds.

**Fig. 5:**
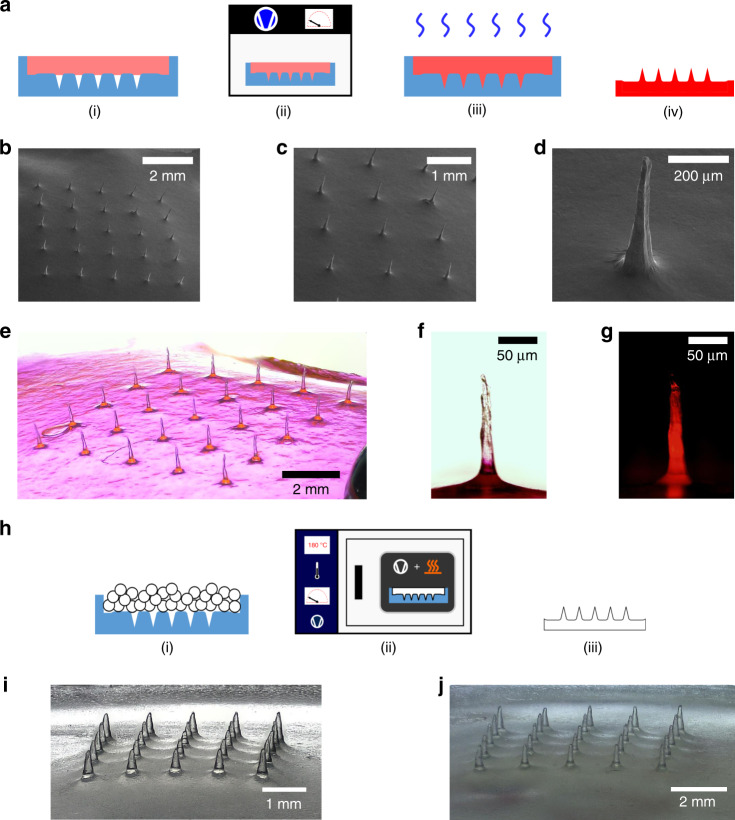
Microneedle fabrication examples. **a** Schematic of fabrication steps of CMC MNAs loaded with Rh B: (i) fill mold with CMC-Rh B solution, (ii) place molds in vacuum chamber to remove air bubbles and fill voids, (iii) dry MNA, (iv) final CMC MNA loaded with Rh B; **b**–**d** SEM images of CMC-Rh B MNAs; **e** optical image of CMC-Rh B MNA; **b** optical image of an individual Rh B-loaded CMC microneedle; **c** optical fluorescence image of a Rh B-loaded CMC microneedle; **h** PLA microneedle casting using mold: (1) mold filling with PLA pellets, (2) placing of mold with pellets in vacuum oven, (3) demolding to obtain PLA MNA; **i** replica molded PLA MNA from a master MNA with a needle-to-needle distance of 1 mm and a fill weight of 500 mg; **j** replica molded PLA MNA from a master MNA with a needle-to-needle distance of 1.5 mm and a fill weight of 500 mg

To test the quality of the fabricated molds, they were used to make MNAs out of two types of polymers as can be seen in Fig. 5. Carboxymethyl cellulose (CMC) MNAs loaded with rhodamine B (Rh B) were fabricated by means of solvent casting (Fig. 5a), whereas PLA MNAs were fabricated using thermal molding (Fig. 5h).

### Skin insertion study

**Fig. 6:**
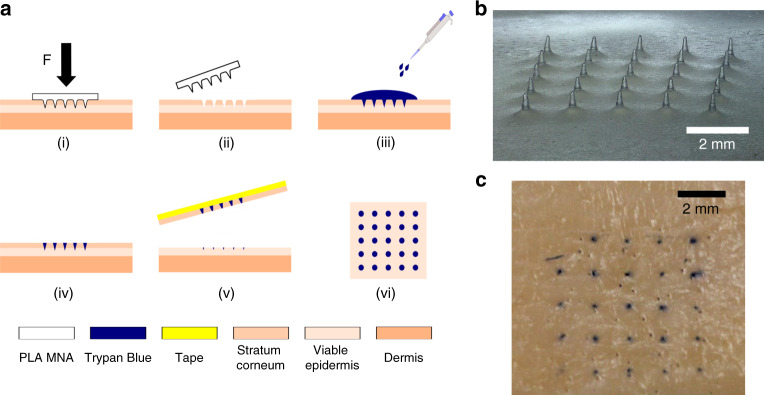
Skin insertion study. **a** Schematic representation of skin insertion test: (i) MNA insertion into the skin applying a force of 30 N for 60 s, (ii) MNA removal, (iii) trypan blue staining left for 120 s, (iv) washing off trypan blue with 0.9% saline solution, (v) tape-stripping of the stratum corneum from the skin, (vi) inspection of the skin sample for trypan blue stains; **b** replica molded PLA MNA (mold master: 1.5 mm needle-to-needle distance and 600 mg fill weight); **c** porcine skin sample after MNA insertion and trypan blue staining

PLA microneedles were fabricated using the newly produced molds (Fig. 6b). A porcine skin insertion study (Fig. 6a) was performed using the PLA MNAs, which showed penetration of the porcine skin as indicated by the trypan blue stains on the needle insertion sites (Fig. 6c).

## Discussion

The resolution of our MNAs compares favorably to previously developed MNAs using low-cost AM methods, such as material extrusion-based FFF and vat photopolymerization-based AM methods. FFF, which is a very low-cost method, suffers from poor resolution in the micron range. To overcome these resolution limitations, Luzuriaga et al.^37^ proposed a two-step fabrication method for printing microneedle arrays, wherein a coarse array is printed in biodegradable PLA which is subsequently etched down to finer features in a potassium hydroxide bath in the second step. This method achieves fabrication of arrays with needle heights of 200–300 μm and a tip sharpness between 1 and 55 μm. However, the actual needle geometry is highly irregular and uneven. Low-cost vat photopolymerization-based methods are therefore preferred when trying to fabricate MNAs. However, attempts to date to directly print needles of sub-millimeter height have generally produced needles with low aspect ratios^45,46^, while the printed needles with higher aspect ratios and good tip sharpness have been in the millimeter range^38^ as opposed to the sub-millimeter range required for painless MNA application. In contrast, our MNAs exhibit sharp tips, sub-millimeter height, as well as a high-aspect ratio.

### Parametric study

In the parametric study, it was shown that sharp microneedles of variable design inputs are printable using a low-cost Form 2 SLA 3D printer. We investigated the effect of varying geometry and print settings on the printed needles. Following a preliminary sweep of materials, Clear Resin was found to provide the most consistent and best quality microneedle geometries. We then examined the effect of layer height on needle quality. It appears to be possible to print needles of acceptable quality using a 25, 50, as well as 100 -µm layer height. Decreasing the layer height, however, resulted in a markedly improved surface finish as edges at an angle to the *z*-axis become smoother. Edge ridge mismatches are a result of an effect called stair-stepping^47^ inherent to printing in layers and become less pronounced with decreasing layer height. While the tip radius of the 100 -µm layer height needles was about 55 µm and therefore theoretically sharp enough to penetrate the skin, the structural integrity was compromised by the relatively large layer height which resulted in more pronounced edge ridge mismatches and therefore increased local stress concentrations. These edge ridge mismatches may also be an issue in demolding of the MNA during fabrication of a silicone mold. The advantages of a greater layer height setting generally are a shorter print time and decreased risk of print failure due a smaller number of individual layers to be cured. As the needles we printed were of short height and therefore consisting of a relatively low number of layers, even with a 25 µm layer height setting, both these advantages were negligible. We therefore decided to continue with the parametric study using a 25 µm layer height as this resulted in a smooth needle surface finish.

The first measured design parameter was the output needle height, which was seen to be lower than the set input height value. While the printed needles were shorter than specified in the virtual model, this appeared to be a predictable factor. Printed needles displayed sharp tips, with tip radii smaller than the printer’s minimum feature resolution which is limited by the 140 µm laser spot size in the *x*–*y* plane. The height discrepancy issue has also been observed in previous studies on vat photopolymerization-based 3D-printed needles. Miller et al.^43^ used the Perfactory III SXGA+instrument system (EnvisionTEC GmbH, Gladbeck, Germany) to print hollow microneedles. The microneedle input dimensions were a triangular base of 1.2 mm edge lengths and a height of 1.5 mm with a 400 μm hollow channel running through it. Measurements obtained showed the printed parts had a base length of 1120 μm, a microneedle height of 1030 μm, and a hollow channel diameter of 375 μm. Differences between dimensions were attributed to the tessellation process by the software which converts the CAD design file to a printable model. After importing the STL file into the print preparation software (PreForm), the print model can be seen to comprise a number of layers equal to dividing its theoretical height by the print layer height setting, e.g., a 3 mm height needle model set to be printed at a 25 µm layer height is divided into 120 layers. It therefore does not appear that tessellation alone can explain the height discrepancy in our case, which is quite substantial. The height discrepancy may be related to the minimum UV dose required for photopolymerization and the way a layer is drawn. UV dose depends on the UV intensity and exposure time with photopolymerization only occurring once a critical UV dose is reached. Light such as UV is diffractive and instead of focusing on a single point will smear out into a blurred spot at the image plane^48^. The intensity of the 405 nm wavelength follows a Gaussian profile, reaching its maximum near the optical axis and falling off laterally^49^. A layer is typically built by a laser tracing the outline of the cross-sectional slice and cross-hatching the space in-between^47^. The distance between the laser traces while hatching is known as hatch spacing and controls the overlap. The closer the hatch spacing, the more overlap exists ensuring a more complete cure. A vertically aligned conical needle during print can be considered a continuously decreasing cross-sectional slice in the *x*–*y* plane and there appears to be a point during printing at which the part stops growing in height. It may be that due to decreasing size of the cross-sectional slice, less overlapping occurs resulting in an overall lesser UV dose for a given point. Due to the hatch pattern, areas close to the center of the slice would not be as affected as areas further away which would begin not receiving the critical UV dose required for polymerization. This process would continue gradually as the cross-sectional area further decreases resulting in round-shaped tips despite the print “failing” before reaching full needle input height. This would also explain why higher aspect ratio needles, which for a given layer will have a lower cross-sectional surface area in the *x*–*y* plane than a needle with a lower aspect ratio, appear to display a slightly increased input-to-output height discrepancy as seen in Fig. 3e. The Form 2 is a closed source system, and we do not know what the exact print instructions and parameters are. Generally, stereolithography is a complex process with over 50 process variables for a resin and part family type^50^, and many of these interacting variables will affect the print outcome of the needle.

A second design parameter measured was the angle *θ*. It is proportional to the aspect ratio for a needle with infinite tip sharpness. This value quantifies the angle between needle sides irrespective of the tip radius. The actual aspect ratio of a real needle will be lower with respect to that of a perfect needle (i.e., a needle of infinite tip sharpness) due to its inherently decreased height resulting from tip rounding. This parameter was relatively proportionate to the input value for needles of greater input height. As needle input height decreased below a value of about 1600 µm, we observed a greater increase of angle *θ* relative to its theoretical value, which indicated a greater than specified needle base diameter, which along with the relative increase of height discrepancy, resulted in needles with low aspect ratios. This can be seen in Fig. 3c, where the smaller needles do not fit in their theoretical 2D outline as indicated by the overdrawn shape. The relative increase grew rapidly as the input height decreased. This observation is most likely related to the minimum feature size in the *x*–*y* plane and the limitations of a 140 -µm laser spot size. The smaller the height for a given aspect ratio, the smaller the base diameter. The base diameter, however, will have a minimum value due to the limitations of the printer’s resolution. This would explain the increase in output to input ratio for *θ* as the needle height decreases. Unlike the gradually decreasing cross-sectional area of the needle, the initial layer of the needle base is a more abrupt appearing feature and thus subject to conventional resolution limitations in the *x–y* plane.

The tip radii of the printed needles are shown to lie between 20 and 40 µm. There appears to be a trend of decreasing tip radius with decreasing needle height. However, taking measurement accuracy and error into account, this difference is deemed not significant. As the needle tips are a result of print “failure” and thus lack of precise control, they are especially subject to needle-to-needle variability. Nevertheless, 20–40 µm tip radii are sufficiently sharp to penetrate the skin. Boehm et al.^42^ used the Perfactory III SXGA+ visible light dynamic mask micro-stereolithography system (EnvisionTEC GmbH, Gladbeck, Germany) to print 1 × 5 arrays of needles which looked to have similar tip sharpness but greater step-size features on the surface compared with the needles we printed. The tip radii we obtained are therefore among the sharpest needles printed using lower-cost printing systems.

The straightness of the needle was also quantified by extracting the needle outline from the images, converting it to a set of data points, and subsequent calculating of the coefficient of determination (R^2^) for the data points for each needle side. A straighter needle will have a *R*^2^ value closer to 1. Calculations found this value to lie in the range of 0.95–0.99 for all needles indicating good straightness of the print.

### Microneedle array fabrication

The parametric study showed that needles of good tip sharpness were directly printable using the Form 2 system. However, for needles not to inflict pain in novel applications, their height should be limited to the sub-millimeter range^18^, and display an adequate aspect ratio to ease insertion. A low aspect ratio may add to mechanical strength of the needle, but can also negatively affect insertion due to its rapidly increasing needle shaft width^51^. In addition, low aspect ratio needles can display poor tip sharpness related to limitations associated with fabrication. Microneedle arrays in previous studies, which were directly printed using the same Form 2 system, did not appear to comprise of features that facilitate ease of insertion. Pere et al.^45^ printed conical and pyramidal needles of 1000 μm input height at an input aspect ratio of 1:1. While no exact dimensions of the printed parts were given, the needles displayed a low aspect ratio and did not appear to be as sharp as the needles we fabricated. Using the same printer, Farias et al.^46^ printed hollow microneedles with a 600 μm height, a 1000 μm base diameter and a tip diameter of 400 μm. The printed needles were shown to have a very low aspect ratio and poor tip sharpness. In order to overcome these issues and be able to fabricate microneedles with a low and controllable height as well as a high-aspect ratio for use as masters in mold making, a two-step fabrication approach was developed. First basins were printed containing a 5 × 5 array of needles with parameters which displayed good results in the parametric study. We chose a “pre-fill” input height of 2.5 mm and an aspect ratio of 4:1, but other values may be chosen as desired. These basins were subsequently filled with UV-curable resin so that sub-millimeter height needles were left exposed. Due to surface tension, the filled needle array basins exhibited a concave meniscus. The inner basin diameter was set to 24 mm, larger than necessary to fit a 5 × 5 needle array of the given needle-to-needle distances used here. This was done to reduce the effect of surface tension during filling. A pre-heat temperature of 80 °C before UV curing was chosen to decrease resin viscosity before the onset of curing. This method yielded MNAs of low sub-millimeter height as can be seen in Fig. 4. Two fill heights (controlled by respective fill weights of 500 and 600 mg) and two needle needle-to-needle distances (1 and 1.5 mm) were investigated, resulting in needles of varying and controllable heights. Needle array basins with a smaller needle-to-needle distance, resulted in a shorter needle height for a given fill weight compared with basins with a greater needle-to-needle distance which is an effect of resin surface tension. As microneedles are mainly developed for application on human/mammalian tissue which itself is quite compliant, it is not expected that a meniscus will greatly affect microneedle insertion performance and may improve mechanical performance under shear loading. The developed method allows for easy fabrication of MNAs with controllable needle-to-needle distance and height. Silicone molds made from these master arrays were used to fabricate CMC MNAs loaded with a fluorescent model drug (Rh B), as well as PLA MNAs. The solvent-cast CMC-Rh B MNAs comprised of very sharp needles (tip radius < 5 μm), as well as displaying a high-aspect ratio (Fig. 5b–f). Fluorescence imaging showed that loading these needles with the Rh B drug model resulted in good drug distribution throughout the MNA and the microneedle tips themselves. The PLA MNAs were not as sharp as the CMC-Rh B MNAs as they were thermally molded and thus are more accurate geometric replicas of the actual MNA master used to make the mold. However, they still displayed good tip sharpness. To investigate whether or not they have sufficient sharpness to penetrate the skin, they were applied in a skin insertion test using porcine ear tissue with subsequent trypan blue staining and tape stripping^52^. The reason for tape stripping is to fully remove the stratum corneum before examining the skin for blue stains indicative of a successful piercing event. If the stratum corneum is not removed, a blue stain dot might be the result of dye residue remaining in a local depression of the skin after microneedle force application which did not actually result in a piercing event. The results showed that fabricated PLA MNAs had a tip sharpness which was capable of piercing porcine skin tissue.

The application of 3D printing in the fabrication of MNA master molds is a simple method which can be adapted by researchers with only basic skills in CAD design required. To date, a wide range of fabrication processes methods have been employed in the production MNA masters, including microelectromechanical systems fabrication and micromachining. MNA masters, such as those made from metal, display good wear properties, enabling long use of the same master. Although use of conventional techniques can be cost-effective and attractive for fabrication of MNA masters, they are often reliant on specialized microfabrication equipment (e.g., milling machines), as well as expertise or access to a person with expertise in using the equipment which may be a limiting factor. Our proposed method offers a viable alternative to previously developed methods. Advantages include the ability to design and fabricate multiple MNA master designs in a short time at a relatively low cost. As desktop 3D printers are suitable for use in the lab, MNA masters may be fabricated in-lab without the need to access a dedicated machining workshop. Larger-scale MNA masters may be produced, the size being limited by the 3D printer’s build area (5 × 5 arrays were used for demonstration purpose in this study). The method is advantageous over other simple methods of MNA master fabrication such as binding of individual needles^6^, which becomes more laborious as patch size and needle density increase and height control may be more challenging than simple filling as we propose here.

Overall this method allows great customizability of MNAs. By changing the design file to be printed, the needle-to-needle distance, overall array size and needle arrangement can be easily altered. There is a discrepancy in actual printed height compared with intended height. This however is not a great issue as one may accurately predict and account for this height loss. The actual final microneedle height is not governed by the printed height, but by the exposed tip height after filling. As this method is quick and simple, researchers may use this print and fill method to fabricate MNAs with varying parameters (e.g., needle arrangement, quantity, density, and height) and find one that best fits their requirements. When the optimum settings have been found, expensive custom molds with these selected parameters may then be bought which are compatible with high volume manufacturing methods such as microinjection molding.

The developed technique allows for fabrication of MNA molds for a variety of applications, including the field of drug delivery which was demonstrated in this study by fabrication of a MNA loaded with a drug model. The drug loading capacity of an individual microneedle is governed by its surface area/volume which in turn is depended on the mold’s dimensional parameters. Therefore, ease of parametric control of molds is advantageous in examining MNA drug loading capacity. In addition, the patch density (number of microneedles per patch) may also be altered which will consequently regulate the total dosage that can be delivered by an MNA^32^. Our method allows for control of parameters by altering the CAD design and in the second step, the fill volume. The drug release profile of microneedles produced from molds fabricated using the proposed method will depend on the physicochemical properties of the chosen polymer. As the release of drugs from an MNA is dependent on the degradation profile of the used polymer and drug diffusion rate from the microneedles, polymers with appropriate molecular weight and degradability can be chosen to control the release of the drug^52^. Given the wide range of polymers that may be used to fabricate MNAs, this technology will be further explored for rapid bolus release and sustained release of drugs. Therefore, this is a simple and promising method which can be adapted for further development of microneedle-based drug delivery systems.

While additive manufacturing generally allows for fabrication of more complex geometries not possible with conventional subtractive manufacturing methods, we deliberately chose to conical-shaped straight needles as our design of choice for several reasons. Conical-shaped needles are extensively used in microneedle research, and are often the preferred design and therefore offering a simple low-cost manufacturing method for that geometry is of great benefit to the researcher. Microneedles with more complex features, such as barbs and overhangs, may offer advantages but may cause issues in replica molding, especially during the demolding step, affecting their suitability for this commonly used fabrication method. Lastly, the resolution of the Form 2 printer poses a limit on more complex microneedle structures and fabricating good quality conical microneedles with sharp tips in itself is challenging (see Supplementary Figs. 1, 2). Desktop SLA 3D printers such as the Form 2 are becoming ubiquitous to the research environment as they allow for rapid prototyping, e.g., printing required parts for experimental rigs. Therefore, these printers may already be available to labs focused on microneedle research, aiding in the adaption of this fabrication technique. Several factors contribute to successful printing of MNAs, including cleanliness of the printer, and previous usage of the resin tank. The methods outlined here could also facilitate the optimization of direct fabrication of MNAs from biocompatible photocurable polymers, which are becoming more readily available.

## Conclusion

We have developed a low-cost, simple, and customizable method for the fabrication of microneedle molds by means of a commonly used, commercially available and affordable SLA 3D printer. A parametric study demonstrated that printing needles with good tip sharpness beyond the printer’s defined resolution limits is possible if the correct print settings are chosen. Printed needle parameters differed from those in the virtual CAD model. Compared with the model, the printed needles were found to be shorter and have a greater than specified aspect ratio. Taking these findings into account, a two-step “Print & Fill” fabrication method was developed with which we were able to rapidly produce microneedle master molds with easily customizable needle parameters. The fabrication method presented is suitable to be directly applied by microneedle researchers in-lab regardless of microfabrication expertise or access to workshops with specialized equipment and allows for production of microneedle arrays with parameters tailored toward their specific requirements.

## Materials and methods

### Materials and equipment

All needle arrays were printed from a UV-curable resin (Clear Resin V4, Formlabs Inc., Somerville, MA, USA) using a desktop SLA 3D printer (Form 2, Formlabs Inc.). The printer employs an inverted stereolithographic process wherein the print is built from bottom up, the laser being directed from below the resin tank and the build head rising an increment after each cured layer. The build volume of the printer is 145 × 145 × 175 mm. The 250 mW laser has a wavelength of 405 nm and a spot size of 140 µm. Depending on material, the printer can provide a theoretical layer thickness as small as 25 µm in the *z*-direction.

### Parametric study

#### CAD design

All designs were 1 × 5 arrays of conically shaped needles with a 1.5 mm needle-to-needle distance. Needle arrays were designed for three different aspect ratios (3:1, 4:1, and 5:1) and for each respective aspect ratio arrays with varying needle height were printed (3.0, 2.8, 2.6, 2.4, 2.2, 2.0, 1.8, 1.6, 1.4, 1.2, 1.0, 0.8, 0.6, 0.4, and 0.2 mm).

#### 3D printing

The 45 different 1 × 5 array CAD design files were exported to STL file format and imported into a print preparation software (PreForm, Formlabs Inc.). The parts were orientated so that the center axis of the needles aligned with the *z*-axis of the printer. The print layer height in the *z*-direction was set to 25 µm for all designs. To investigate the effect of print layer height, needles with an aspect ratio 4:1 and heights ranging from 3.0 to 2.0 mm were printed in addition to the previous set of arrays.

#### Post-print treatment

After printing, the needle arrays were washed in an isopropanol (IPA) bath for 20 min (Form Wash, Formlabs Inc.). During washing, the IPA was agitated using a magnetically coupled impeller. After washing, the needles were post-cured for an hour by means of UV LEDs (*λ* = 405 nm) at a temperature of 60 °C (Form Cure, Formlabs Inc.).

#### Needle inspection

After initial visual inspection, needles with a set input heights of 200 and 400 µm were deemed of insufficient quality for parametric analysis. For the remaining needles, parameter dimensions were approximated by performing an analysis on digital images of samples taken with a stereomicroscope (SZN-T, Optika Srl., Ponteranica, BG, Italy) using MATLAB (MATLAB R2018b, MathWorks Inc., Natick, MA, USA).

### “Print & Fill” microneedle mold fabrication

#### Needle array basin design

A 3D model for a 5 × 5 needle array basin was developed using CAD software (Autodesk Inventor Professional 2017, Autodesk Inc., San Rafael, CA, USA). The needles were designed to be conical in shape, having a height of 2.5 mm with a 625 µm base diameter, resulting in a height aspect ratio of 4:1. The array was situated within a basin (24 mm inner diameter) with a wall thickness and height of both 1 mm. Two types of needle array basins were designed, one with a 1 mm and another with a 1.5 mm needle-to-needle distance.

#### 3D printing

The CAD model of the needle array basins were exported to STL file format and imported to a print preparation software (PreForm, Formlabs Inc.). A needle array basin was orientated so that the center axis of the needles aligned with the *z*-axis of the printer. The print layer height in the *z*-direction was set to 25 µm, and support structures were added to the bottom of the basin. The model with associated print settings was then uploaded to the printer.

#### Post-print treatment

After printing, a needle array basin was washed in an IPA bath for 20 min (Form Wash, Formlabs Inc.). During washing, the IPA was agitated using a magnetically coupled impeller. After washing, the basin was post-cured for an hour by means of UV LEDs (*λ* = 405 nm) at a temperature of 60 °C (Form Cure, Formlabs Inc.).

#### Basin filling

In the next step, a UV-curable resin (Grey Resin V4, Formlabs Inc.), used in this study for contrast as opposed to clear resin, was pipetted into the basin leaving only a sub-millimeter height needle tip exposed. The basins were filled with both 500 and 600 mg of resin to obtain different needle heights. The filled basins were then UV cured (*λ* = 405 nm).

#### Microneedle replica molding

A needle array basin, which now had exposed microneedles and therefore could be considered an MNA, was dip-coated in a liquid agent (Inhibit X, Mann Formulated Products, Inc., Macungie, PA, USA) which protects against cure inhibition when pouring platinum-cure silicones. A mold release (Ease Release 200, Mann Formulated Products, Inc.) was sprayed onto the MNA. A mold of the MNA was then made using a platinum-cure silicone (PlatSil 7315, Mouldlife, Bury St Edmunds, Suffolk, UK). After demolding, a silicone microneedle mold was obtained.

#### Polymer MNA fabrication

##### CMC MNAs loaded with Rh B

Rh B-loaded CMC MNAs were fabricated from a silicone mold by means of solvent casting. In all, 5% (w/v) sodium carboxymethyl cellulose (M_W_ ~ 250,000, Sigma-Aldrich, St. Louis, MO, USA) was added to deionized water, pre-mixed on a vortex mixer, and left in a heated water bath at 60 °C for an hour. Rh B was then added to the solution at a mass concentration of 0.1 mg/ml. The solution was again mixed on a vortex mixer and left in a heated water bath at 60 °C for an hour. Next the solution was centrifuged at 6000 rpm for 5 min (FC5706, OHAUS Corporation, Parsippany, NJ, USA) followed by a mixing stage in a planetary mixer (AR-250, THINKY U.S.A., Laguna Hills, CA, USA) at 1500 rpm for 90 s. The solution was then transferred into previously fabricated silicone MNA molds and placed under vacuum for degassing and filling voids. The MNA was then dried until all the water was evaporated and Rh B-loaded CMC MNAs were obtained. Fluorescence imaging of the Rh B-loaded CMC MNAs was performed on a fluorescence microscope (Olympus Corporation, Shinjuku, Tokyo, Japan).

##### PLA MNAs

An MNA was fabricated from a silicone mold using a biocompatible PLA (4032D, NatureWorks LLC, Minnetonka, MN, USA) by means of vacuum molding in a an oven (3608-6CE, Thermo Fisher Scientific Inc., Waltham, MA, USA). The material has a quoted tensile strength of 103 MPa in the transverse direction and 145 MPa in the machine direction as tested according to ASTM Test Method D882. The fabricated mold was filled with the PLA pellets and placed in the vacuum oven at a temperature 180 °C and a pressure of 98.2 kPa for 20 h ± 4 h until the PLA had filled the mold cavities and all air bubbles were removed.

#### Skin insertion testing

A fabricated PLA MNA was inserted into porcine ear tissue (35-day-old Large-White and Landrace hybrid sow with MAXGRO semen) at a force of 30 N for 60 s using a handheld force gauge (Sauter FK100, Sauter GmbH, Balingen, Germany). A solution of 0.4 wt% trypan blue (Sigma-Aldrich, St. Louis, MO, USA) was applied on the insertion area, left for 120 s, and then washed off with 0.9% saline solution (Lennox, Dublin, Ireland). Tape stripping using a generic commercial sellotape was performed to remove the stratum corneum after which the skin was inspected for successful insertion of microneedles.

## Supplementary information

Supplemental Figure S1

Supplemental Figure S2

Supplemental Figure S3

Supplementary Information
