# Supplementary figures and images for: Simple and customizable method for fabrication of high-aspect ratio microneedle molds using low-cost 3D printing
Source: Microsyst Nanoeng. 2019 Sep 9;5:42. doi: 10.1038/s41378-019-0088-8 (PMC6799892; doi:10.1038/s41378-019-0088-8)

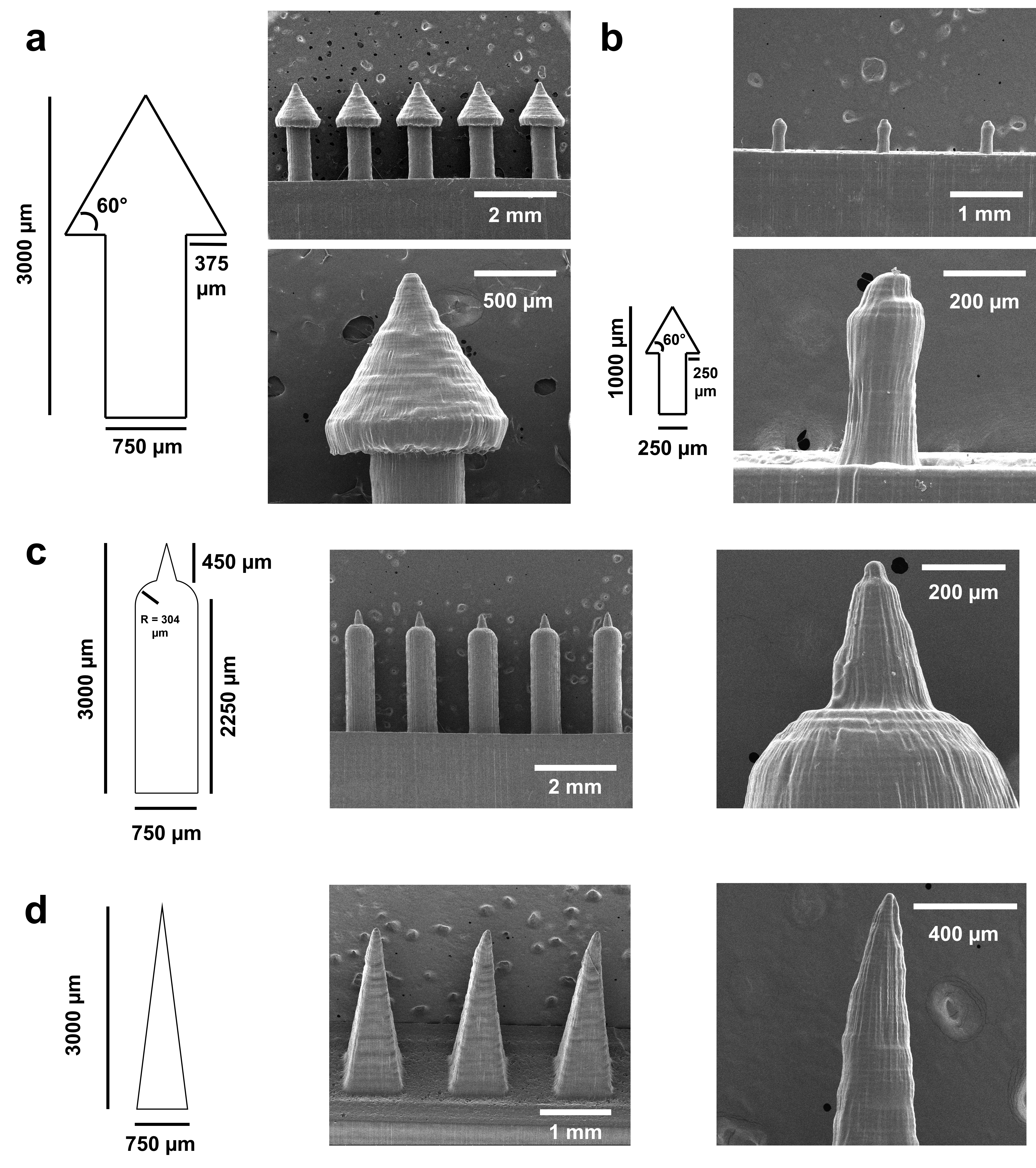

Supplement: Supplementary file 1 — Supplemental Figure S1 [file 41378_2019_88_MOESM1_ESM.tif]

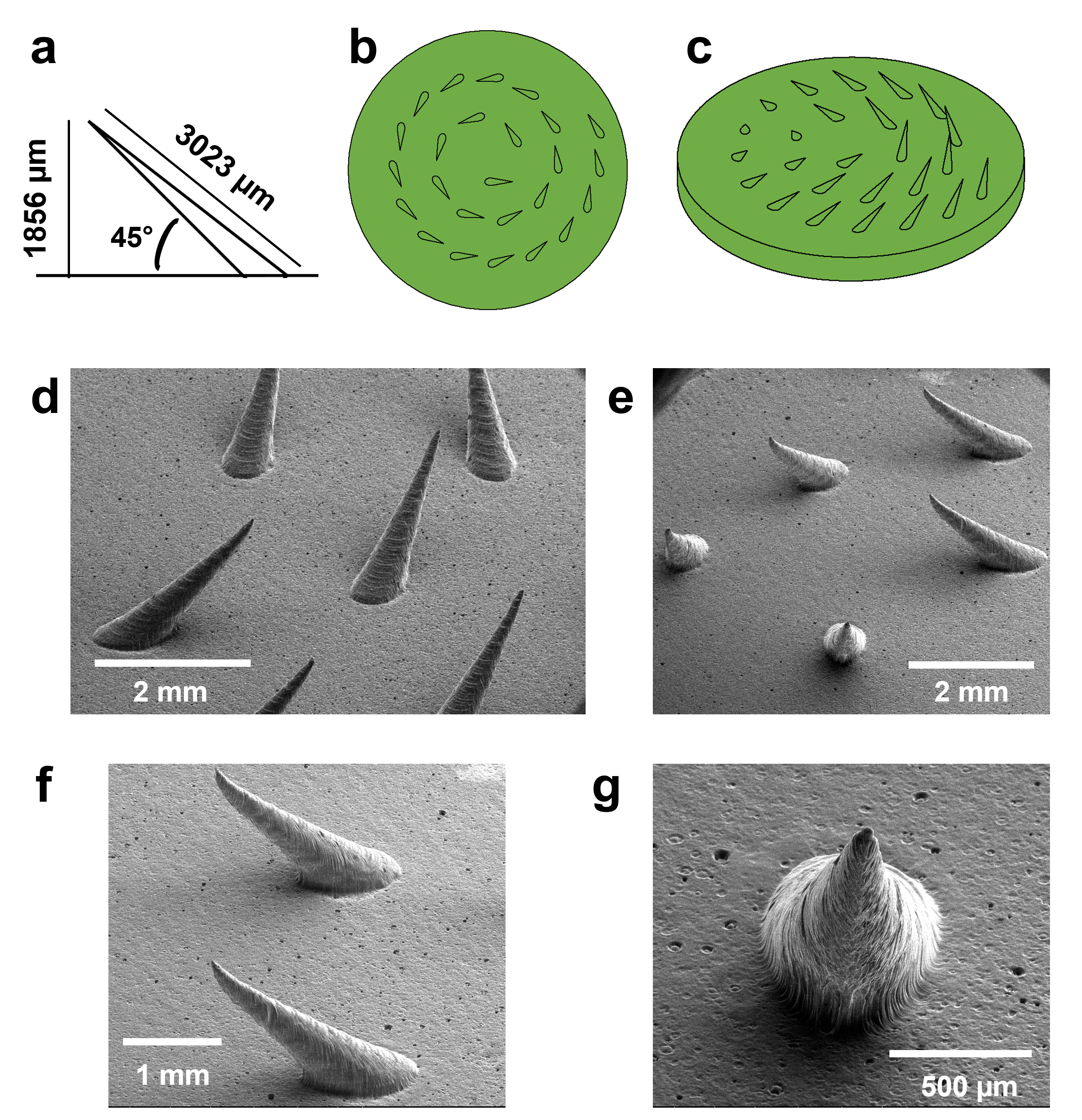

Supplement: Supplementary file 2 — Supplemental Figure S2 [file 41378_2019_88_MOESM2_ESM.tif]

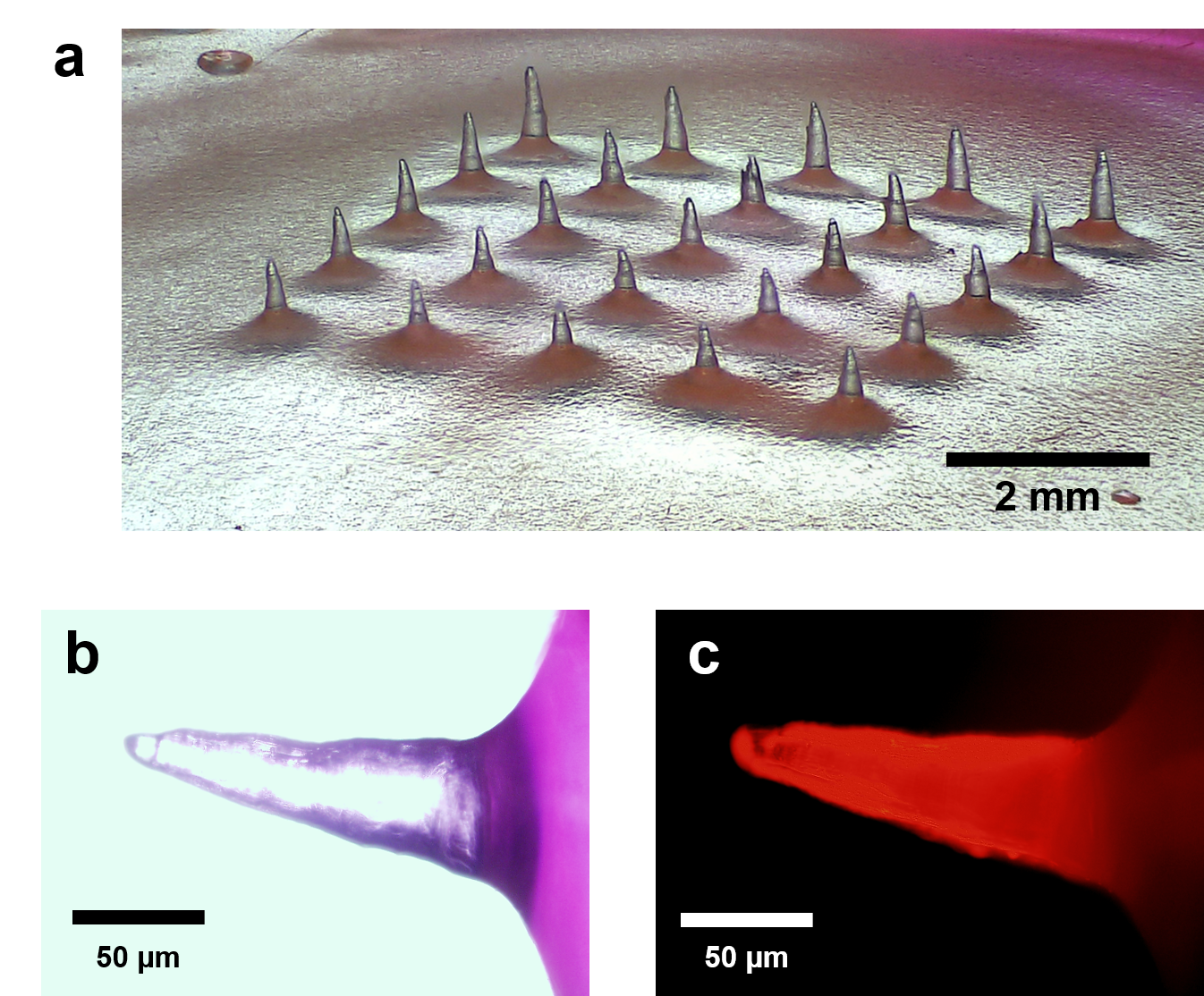

Supplement: Supplementary file 3 — Supplemental Figure S3 [file 41378_2019_88_MOESM3_ESM.tif]
