# Supplementary material for: Simple and customizable method for fabrication of high-aspect ratio microneedle molds using low-cost 3D printing
Source: Microsyst Nanoeng. 2019 Sep 9;5:42. doi: 10.1038/s41378-019-0088-8 (PMC6799892; doi:10.1038/s41378-019-0088-8)
Supplement: Supplementary file 4 — Supplementary Information [file 41378_2019_88_MOESM4_ESM.docx]

**Supplementary Information**

**Additional Geometries**

Additional more complex needle shapes and array arrangements were printed on a Form 2 printer using Clear Resin V4 at a layer height setting of 25 µm.

**Needle Shapes**

**Figure S1 – Microneedle Geometries.** **a** Mushroom-shaped needle geometry printed at a 3 mm input height; **b** Mushroom-shaped needle geometry printed at a 1 mm input height; **c** Turret needle geometry printed at a 3 mm input height; **d** Square-based pyramid needle geometry printed at a 3 mm input height

**Needle Array Arrangement**

An alternative needle array was printed comprising of 25 angled needles in a spiral arrangement.


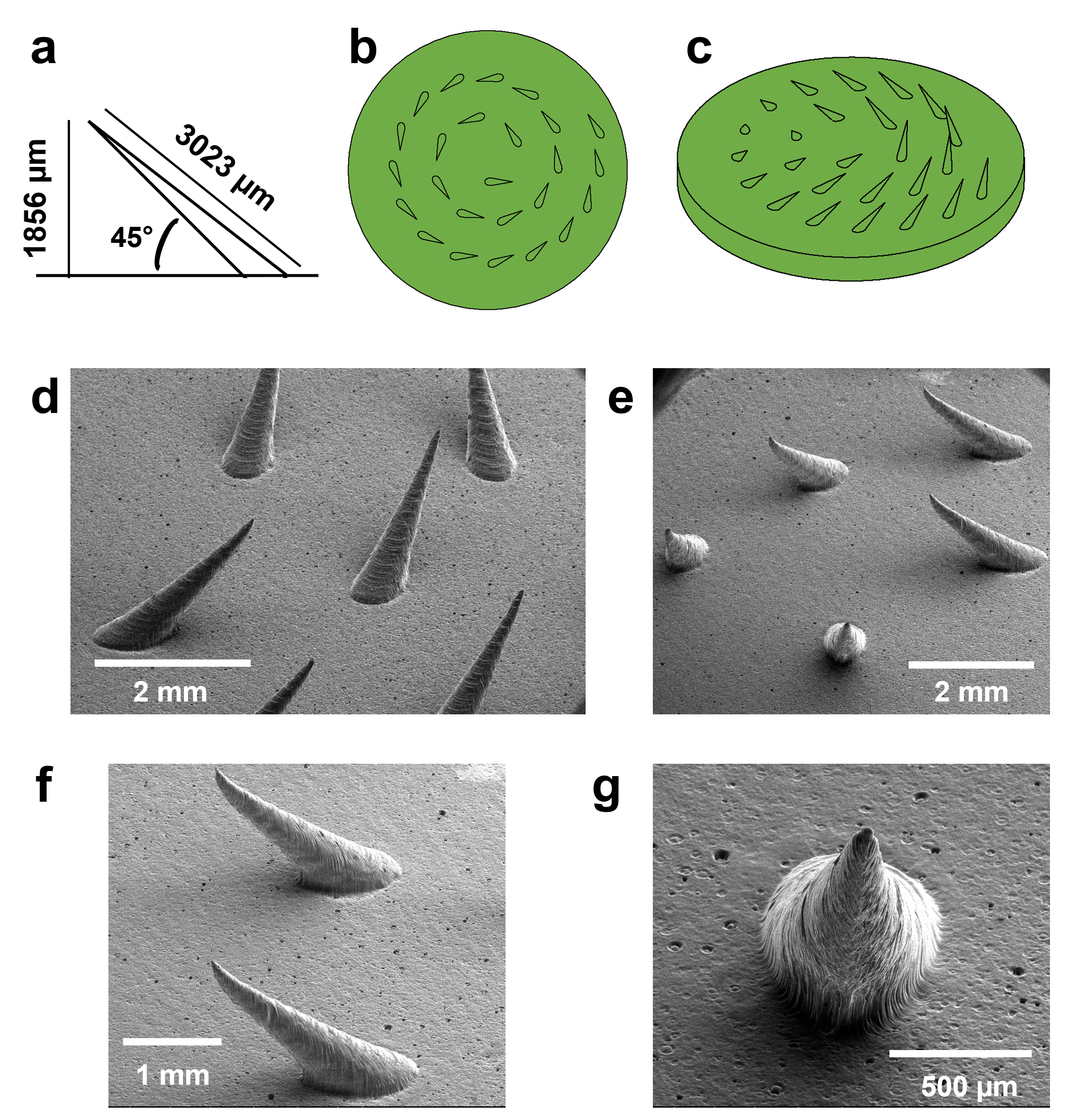


**Figure S2 – Spiral Screw Patch.** **a** Schematic of individual needle input dimensions; **b & c** CAD drawings of spiral screw patch design; **d – g** SEM images of printed spiral screw patch

**Polyvinylpyrrolidone (PVP) MNAs loaded with Rh B**

Rh B-loaded PVP MNAs were fabricated from a silicone mold by means of solvent casting.

**Fabrication Steps**

30 % (w / v) PVP (**M_W_ ~ 40,000,** Sigma-Aldrich, St. Louis, MO, USA) was added to deionized water, mixed in a planetary mixer (AR-250, THINKY U.S.A., Laguna Hills, CA, USA) at 1500 rpm for 90 s and left in a heated water bath at 60 °C for an hour. Rh B was then added to the solution at a mass concentration of 0.1 mg / ml. The solution was again mixed in a planetary mixer at 1500 rpm for 90 s left in a heated water bath at 60 °C until it was used for solvent casting. The solution was then poured into previously fabricated silicone MNA molds and placed in under vacuum for degassing and filling voids. The MNA was then dried until all the water evaporated and Rh B-loaded PVP MNAs are obtained. Fluorescence imaging was performed on a fluorescence microscope (Olympus Corporation, Shinjuku, Tokyo, Japan).


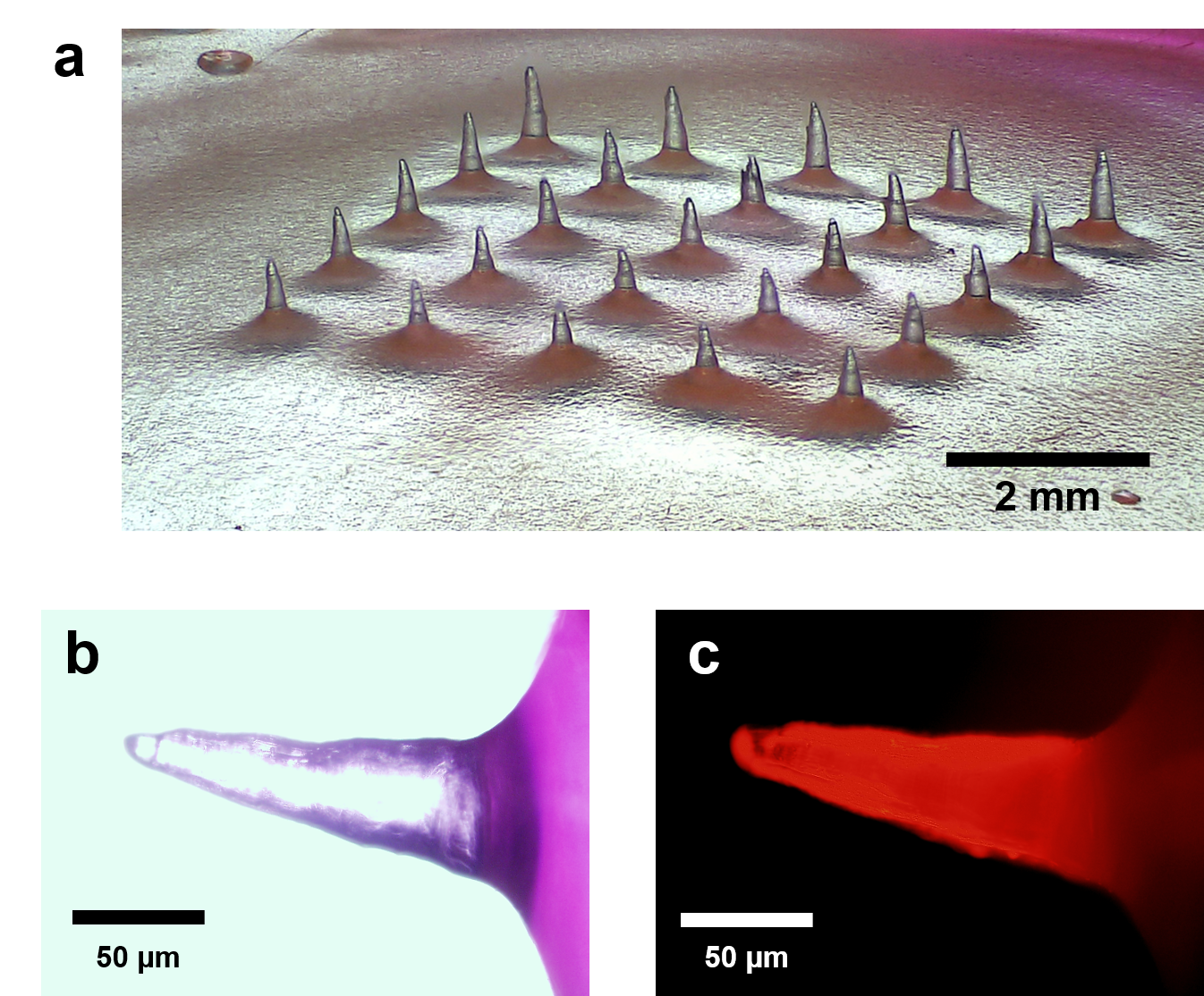


**Figure S3 – PVP-Rh B MNAs.** **a** Optical Image of a PVP MNA loaded with Rh B; **b** Optical image of an individual Rh B-loaded PVP microneedle **c** Optical fluorescence image of a Rh B-loaded PVP microneedle
